# Mark-release-recapture meets Species Distribution Models: Identifying micro-habitats of grassland butterflies in agricultural landscapes

**DOI:** 10.1371/journal.pone.0207052

**Published:** 2018-11-28

**Authors:** Jan C. Habel, Mike Teucher, Dennis Rödder

**Affiliations:** 1 Terrestrial Ecology Research Group, Department of Ecology and Ecosystem Management, School of Life Sciences Weihenstephan, Technische Universität München, Freising, Germany; 2 Department of Remote Sensing and Cartography, Institute of Geosciences and Geography, Universität Halle, Halle, Germany; 3 Zoologisches Forschungsmuseum Alexander Koenig, Bonn, Germany; Charles University, CZECH REPUBLIC

## Abstract

Habitat demands and species mobility strongly determine the occurrence of species. Sedentary species with specific habitat requirements are assumed to occur more patchy than mobile habitat generalist species, and thus suffer stronger under habitat fragmentation and habitat deterioration. In this study we measured dispersal and habitat preference of three selected butterfly species using mark-release-recapture technique. We used data on species abundance to calculate Species Distribution Models based on high-resolution aerial photographs taken using RGB / NIR cameras mounted on a UAV. We found that microhabitats for species with specific habitat requirements occur spatially restricted. In contrast, suitable habitats are more interconnected and widespread for mobile habitat generalists. Our models indicate that even managed grassland sites have comparatively little habitat quality, while road verges provide high quality micro-habitats. In addition, dispersal was more restricted for specialist butterfly species, and higher for the two other butterfly species with less ecological specialisation. This study shows synergies arising when combining ecological data with high precision aerial pictures and Species Distribution Models, to identify micro-habitats for butterflies. This approach might be suitable to identify and conserve high quality habitats, and to improve nature conservation at the ground.

## Introduction

Habitat configuration and habitat quality significantly influence the occurrence and persistence of species [1]. Today, many remaining habitats are small and geographically isolated from each other, as most semi-natural calcareous grasslands across agricultural landscapes of Central Europe [2]. Recent studies showed that continuing fragmentation of remaining habitats and further intensification of agriculture in between lead to barriers for many species [3]. Furthermore, habitat quality in remaining habitat patches becomes deteriorated due to the influx of pesticides, pollutants and nitrogen [4–5]. These factors drive species losses and the reduction of species´ abundances, as identified for grassland butterfly species, particularly on calcareous grasslands [6]. This situation becomes even more precarious due to the fact that most remaining habitats are small and geographically isolated and many butterfly species show sedentary dispersal behaviour, with limited migrations across the landscape matrix [7–8]. This may accelerate local extinctions with the result that many potential suitable habitats remain `un-occupied´ [9]. Species demanding specific habitat requirements and with restricted dispersal suffer in particular under habitat fragmentation and the deterioration of habitat quality [10].

In nature conservation, especially high quality habitats considered a priority for habitat management and the protection of sites. Remote sensing techniques and high resolution aerial photographs may help to identify such high value sites. In this study we measure the availability of suitable microhabitats for three butterfly species differing in habitat specialisation and dispersal behaviour [11–20]. To identify these microhabitats we assessed species abundances at points which were randomly set. In parallel, we performed mark-release-recapture techniques to quantify the dispersal behaviour of our targeted species. To identify the spatial composition availability of microhabitats we used high-resolution aerial pictures taken from a UAV equipped by RGB / NIR cameras. Subsequently, Species Distribution Models were run (i) based on species’ presence/absence, and (ii) species abundance of individuals of the respective species reflecting home range preferences. We will interpret our model-outcome against the background of species´ specific dispersal behaviour obtained from our mark-release-recapture data. Based on our data we test the following hypothesis: (i) Suitable habitats for specialist butterflies occur patchy and are mainly restricted to managed sites if compared with habitat generalist butterflies; (ii) Specialist species show more restricted dispersal behaviour if compared with generalist species.

## Material and methods

### Study species

Our selected butterfly species occur in high population densities across our study area. *Melanargia galathea* is a common habitat generalist found at flower-rich fresh to dry grassland sites, which uses a comparatively broad ecological niche and occurs in high densities in extensively used grasslands, along road verges and forest edges [11–14]. Caterpillars feed on different species of grasses [11–14]. The species can be classified as a mobile butterfly [13], see also previous MRR studies [15]). The two other butterflies, *Erebia medusa* and *Coenonympha arcania* are more specialised than *M*. *galathea*. *Erebia medusa* occurs at nutrient-poor semi-open habitats like extensively used meadows and young fallows, fens, marshy forest meadows and clear cuttings and needs wind protecting structures as forest edges or hedge rows [16]. Its larval food plants are grasses (*Poaceae* mainly) [12]. The butterfly species requires specific open sites within the swards for oviposition. Eggs are deposited mostly more than 10 cm above ground so that moving before the eclosion of the caterpillars (i.e. before mid- to end-June) will destroy the next generation [16]. Consequently, the species decreases strongly on the landscape level due to agricultural intensification and afforestation [17]. This butterfly shows a sedentary dispersal behaviour [18]. Similarly specialised and sedentary is *Coenonympha arcania*. This butterfly occurs restricted to structure-rich, nutrient-poor grasslands, especially near forests, clearings in flood plain woodlands, but also in light sunny forests with a lot of understory [13]. Its larvae feed on various grass species. *Coenonympha arcania* is strongly declining in some regions of Central Europe due to the vanishing of extensively used fresh grasslands and young heterogeneous fallow land (mosaic of woody-open structures) [17, 19–20].

### Study area

As study area we selected the managed calcareous grassland site `Dietersheimer Brenne´ (45ha), surrounded by agricultural land, forest, roads and paths. Our study area is located south of the city Freising (48.29N, 11.68E). The `Dietersheimer Brenne´ is characterised by high intensity of solar radiation, high temperature peaks, and dry climatic conditions [21]. Mesobromion is the dominating vegetation formation which consists of a species rich flora and fauna, including many rare and protected taxa [2]. Current conservation management antagonise the reforestation of this grassland site.

### Aerial pictures

Aerial pictures were taken with a UAV (DJI Phantom 3 Professional) equipped with a 3-axis gimbal, standard DJI RGB camera (1/2.3” CMOS sensor), and a modified DJI camera for acquisition of Near Infrared Imagery (3.97mm f/2.8 82° HFOV Red + NIR channel) [22]. The flight was conducted in August 2017 at a height of 60 m aboveground using an orthogonal camera attachment with 12MP resolution, 94° field of view (FOV), 20mm focus equivalent and 2s time lapse interval while flying with 5 m/s. Flight path was prepared using the software Litchi for DJI Phantom [23], with stop and turn curve mode settings. Missions were prepared with minimum 50% overlap between consecutive pictures and between neighbouring flight lines to minimize lens distortion effects on results. In total, four flight lines in one flight mission (15 min) were conducted with each flight line of 250 m length composed as rectangular shape. For NIR photographs we used the MAPIR Camera Reflectance Calibration Ground Target Package (V1) [24]. In total 1825 usable pictures were taken for RGB and NIR. Single aerial pictures were post-processed using AgiSoft Photoscan Professional software with high-quality dense cloud processing and mesh construction settings. The calculated Digital Surface Model (DSM) was used in Photoscan Professional to create minimal distorted orthomosaic, due to undulating relief in the study area. NIR aerial imagery was reflectance calibrated using MAPIR Camera Control Application (MCC) and processed with identical settings. Based on geocoded imagery and visual verification of accuracy processed imagery was exported as orthomosaic in geotif raster file format with 0.02 m cell size and resampled to 0.2 m cell size for subsequent use in species distribution modelling. The processed orthomosaic achieved absolute projection error below 3m for XY-errors, due to missing high precision ground references. Aerial pictures were decomposed into RGB + NIR channels, which were used as input variables for the habitat suitability models. In preparation to modelling approach a Digital Surface Model DSM was created, too, using 0.04m resolution and clipped and resampled to same extent as orthomosaic using bilinear interpolation. For both, occupied and unoccupied plant sites we approved all locations again in the field using the previously taken GPS coordinates which are now combined with the generated and georeferenced orthomosaic. As all point localities coincide with our field sites we used all data for further habitat suitability models.

### Mark-release-recapture

Mark-release-recapture was conducted inside the managed grassland site `Dietersheimer Brenne´. Points for butterfly capturing were randomly set across the study site (with 65 points in 2014 and 20 points in 2015 and 2017). Point selection was done using “random points in layer bounds” in QGIS 2.18 [25]. At each of these points we captured the targeted butterfly species for 10 min during its flight season. Data were collected during the flight period of the respective study species. Capturing of butterflies took place between 10am and 4pm during days with sunny weather conditions and air temperatures above 20°C. Butterflies were netted in the field and marked with a running number with a water proof pen. For each individual we recorded date, time and sex. Individuals which were already marked were classified as recaptures. For all species we considered the number of individuals marked at each study point, as well as the proportion of migrating individuals (in general, and sex-wise). Furthermore we analysed mean, minimum and maximum distances across all individuals, and for both sexes, separately. Radius of action was calculated using minimum bounding geometries in QGIS 2.18 [25] covering sampling plots were marked individuals were captured within one season. All raw data are provided in S1 and S2 Appendix.

### Species distribution models

For SDM development we pooled the observations at the randomly selected points for each species. As the datasets also allow to quantify relative abundances at the sampled plots we followed two different approaches: we (1) run standard SDMs using only one observation per plot if the species was generally present and (2) added each observation to the training dataset for SDM development, i.e. including multiple observations at the same site if present. The latter approach weights the modelled preference of the species in environmental space according to the measured abundance in the field, while the first approach assesses the relative suitability of a given site based on the general distribution of the species in environmental space across occupied sites throughout the study area irrespective of local abundances. The movement information of each species as described by our mark-release-recapture data was used to assess the spatial distribution and accessibility of suitable micro-habitats.

The potential micro-habitats of the study species were quantified based on the remote sensing variables derived from areal pictures taken by the UAV, i.e. the DSM, the red, green and blue band of the aerial photo as well as the normalized differentiated vegetation index (NDVI; calculated as NDVI = (NIR—red)/(NIR + red)). These variables are suitable to describe both the general surface cover and its spatial structure as well as to discriminate different vegetation types. All environmental variables had a pairwise inter-correlation of R^2^ < 0.75 and can hence be treated as predominately independent variables, as required by the SDM algorithm.

As presence-pseudoabsence algorithm, we chose Maxent 3.4.1. as machine learning technique [26–27]. Maxent follows a maximum entropy approach and derives the potential distribution of a species from environmental conditions at its presence locations and the available environmental space within the study region. Testing various combinations of environmental variables and features thereof, Maxent uses a random walk methodology to derive the best suited model to describe the distribution of species’ occurrences without making too complex assumptions. As environmental background to select pseudo-absences, we defined an area defined by a buffer of 50 m enclosing the sample plots. Restricting the background is necessary to account for dispersal limitations of the target species as pseudo-absences are ideally selected from potentially accessible sites. As the results of single Maxent SDMs due to the machine-learning approach and data splits for model evaluation may provide multiple alternative best fitting models, multiple SDMs were computed. Therefore, for model evaluation across our both approaches we computed 100 single SDMs, each splitting the species records in 80% used for model training and 20% used for model evaluation based on the area under the receiver operating characteristic curve (AUC; [28]). The averages across all single SDM projections using the Cloglog output format were used for further processing and rescaled based on the minimum training presence threshold. Potential extrapolation beyond the training range of the variables was quantified using multivariate environmental similarity surfaces (MESS; [29]), which allow the identification of those sites characterized by environmental conditions beyond the training conditions of the SDMs. These sites are accompanied by a high prediction uncertainty and were omitted in subsequent analyses.

## Results

### Mark-release-recapture

The average radius of action (means across all years, 2014, 2015 and 2017) varied strongly among the three species analysed (χ^2^ = 59.4, P < 0.05). We found strongest dispersal for *M*. *galathea* (number of recaptured individuals, n = 85, 70.3m (SE = 3.4) (ranging from 18-147m), intermediate dispersal for *E*. *medusa* (n = 101) 52.9m (SE = 3.5), ranging from 8–150m), and most restricted dispersal behaviour for *C*. *arcania* (n = 100, 34.5m (SE = 3.0), ranging from 5–138m).

### Habitat suitability models / Species distribution models

From a statistical point of view both approaches revealed good results in terms of AUC scores (AUC_unique, training_: range = 0.815–0.858; AUC_unique, test_: range = 0.785–0.829; AUC_abundance, training_: range = 0.822–0.900; AUC_abundance, test_: range = 0.819–0.896; Table 1). Across all models, RGB Red had the highest variable contribution, followed by DSM in most cases (Table 1). RGB Green had in all models the lowest explanative power. The same general pattern of variable contributions was suggested when using a permutation procedure. Within the projection area of the SDMs, the MESS analyses revealed no sites which would require model extrapolation beyond the training range. Hence, MESS results are not shown.

**Table 1:** Summary of SDMs developed based on unique observations per grid cell and abundance data.

Species		*C*. *arcania*		*M*. *galathea*		*E*. *medusa*	
Set		unique	abundance	unique	abundance	unique	abundance
	Number of training samples	32	702	62	1649	54	624
	Number of test samples	7	175	15	412	13	156
	Training AUC	0.858	0.900	0.827	0.822	0.815	0.863
	Test AUC	0.829	0.896	0.800	0.819	0.785	0.855
**Variable contribution**							
	DSM	30.6	34.2	17.7	27.1	16.9	16.2
	NDVI	6.9	11.4	4.3	6.4	4.4	19.5
	RGB Blue	14.3	18.6	2.2	6.7	2.5	15.3
	RGB Green	0.9	0.6	1.5	1.6	1.3	3.8
	RGB Red	47.3	35.2	74.4	58.3	74.8	45.2
**Permutation importance**							
	DSM	51.2	33.5	11.8	21.9	16.3	13.7
	NDVI	10.3	13.8	2.4	8.3	4.3	18.7
	RGB Blue	3.8	6.0	2.4	7.7	3.5	12.9
	RGB Green	2.5	1.8	6.5	5.0	4.3	3.9
	RGB Red	32.2	45.0	77.0	57.1	71.5	50.8
**Presence / absence thresholds (Cloglog)**							
	Minimum training presence	0.0776	0.0378	0.0792	0.1376	0.1058	0.0902
	10 percentile training presence	0.2626	0.2469	0.3585	0.362	0.3728	0.3143

**Fig 1:**
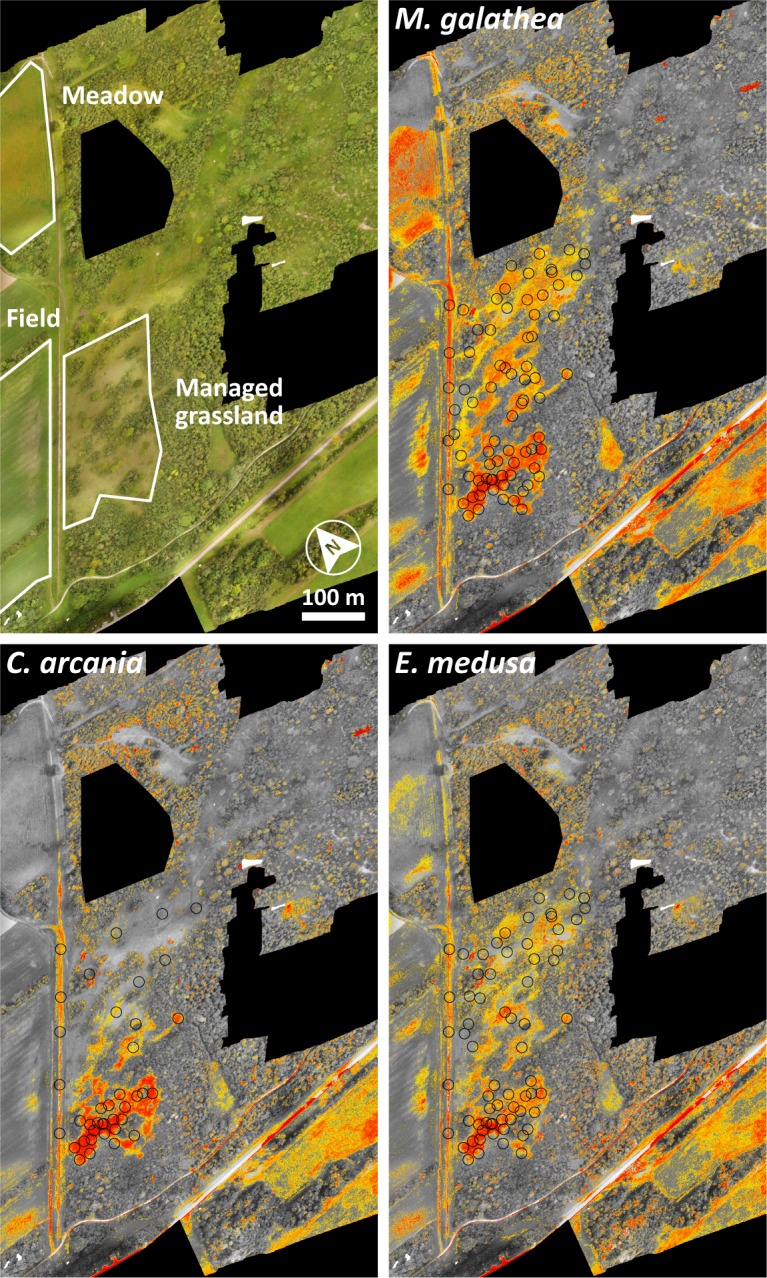
Study area and potential distribution of *Melanargia galathea*, *Erebia medusa* and *Coenonympha arcania*. Warmer colours suggest higher environmental suitability. Circles indicate sampling locations where mark-release-recapture was conducted. Black areas were not covered by UAV flights.

Comparing the results obtained with single observations per grid cell most open areas within the study area are suitable for all three species. However, accounting for local abundances the model suggests a much more patchy occupancy of micro habitats reflecting conditions of homerange cores, wherein *M*. *galathea* may occupy a much larger area then *E*. *medusa* and *C*. *arcania* (Fig 1). The results suggested by the SDMs are in concert with the expectations on the habitat requirements and the availability of species´ specific microhabitat niche.

## Discussion

### Ecological requirements and dispersal

Our models show that the degree of ecological specialization is reflected by the distribution of suitable habitats. Most suitable habitats are identified for *Melanargia galathea*, while suitable habitats occur more patchy for the two species *Erebia medusa* and *Cynonympha arcania*. Our data are congruent with previous studies on butterfly species supporting that increasing habitat specialization lead to the reduction of the availability of suitable habitats, and thus reduces species´ occurrences [30]. Studies showed that such specialized species are only hardly able to respond on environmental changes [31]. Butterflies on the need for specific micro-habitat structures, specific larval food plants and/or specific nectar sources suffer in particular under recent landscape homogenization [13, 32]. In addition, specialist species are characterized by restricted dispersal behavior [13]. This combination of patchy occurrences and restricted movement might aggravate potential negative effects arising from habitat fragmentation and the deterioration of habitat quality. Subsequently, such taxa are assumed to suffer more under demographic and environmental stochasticity than species equipped with higher ecological plasticity [33–35]. Restricted movement and the fact that most high-quality habitat patches are small and geographically isolated from each other causes a silent vanishing of many taxa across our landscape, and thus, many suitable habitats remain unoccupied [9, 36].

Our models identified a high habitat quality along roads and paths for all three model species. This might arise from occasional disturbances which may produce suitable habitat structures for butterflies. These habitats along linear pathways may also serve as suitable corridors for the migration of butterflies, and its exchange among suitable habitats [37–39]. We argue that, particularly habitat specialists with restricted dispersal behavior such as *Erebia medusa* and *Cynonympha arcania*, may profit from such surrogate habitats and corridors which may help to increase landscape permeability.

### Habitat quality

Our study area consists of a mosaic of managed extensively used grassland (Mesobromion), forests, agricultural fields, roads and paths. Our SDMs show a high habitat quality for all three study species along roads and paths. In contrast, major parts of the managed grassland are of rather low quality, despite land management. This may have various reasons: Atmospheric nitrogen deposition may diminish habitat quality, particularly of nitrogen-limited ecosystems such as calcareous grasslands [40–41]. Previous studies showed that typical grassland butterflies suffer under current atmospheric nitrogen deposition which changes vegetation structures. Atmospheric nitrogen increase growth rates of vegetation and thus may lead to higher standing grasses and denser vegetation; This affects microclimatic conditions (increasing shadow and subsequent higher humidity and lower temperatures), and the outcompete of warm-loving typical calcareous grassland species. This either reduces micro-habitat quality or may completely lead to the vanishing of specific grassland species, with detrimental effects on the successful larval development of butterfly species [6]. This particularly affects butterfly species which rely on typical grassland plant species. This trend of deterioration of habitat quality is currently observable in many nature reserves of the EU, which are embedded in agricultural landscapes [40–41]. The fact that road verges and paths, characterized by occasional disturbances and thus a rather open vegetation might be another evidence, that atmospheric nitrogen causes the deterioration of (undisturbed) grassland habitats. Another potential reason may arise from management strategies itself: previous studies showed that specialized butterfly species may respond highly sensitive on marginal changes in habitat structures [42–44]. Thus, conservation management focusing on other taxa such as orchids may lead to the vanishing of endangered arthropod populations [45–46].

### Conclusion

With our data we approve both hypothesis raised at the end of our introduction. The combination of detailed field observations (such as MRR) with high resolution aerial pictures allows the projection of suitable micro-habitats as well as the quantification of micro-habitats used during transitional movement or as home range cores. This approach underlines that classical field observations provide ecological and behavior (as habitat use and dispersal behavior) needed. Results obtained from SDMs can only become interpreted against the background of field observations, such as dispersal behavior. For example, suitable habitat patches might exist across the landscape, however, its (re)colonisation by a butterfly species might be predicted to be unlikely due to its restricted dispersal behavior, according to MRR data. Important synergies may arise from both approaches and allow the combination of two spatial scales (from microhabitat to landscape scale). Advantages of the UAV-acquired imagery compared to very high-resolution (VHR) imagery are the flexibility for the date of acquisition to ensure optimal differentiation between different habitat types. Furthermore, the simultaneously acquired data were necessary for correct SDM calculations. Although the mentioned missing high precision ground reference points reduce absolute positioning accuracy the usage of VHR imagery (i.e. Quickbird, Pleiades) could not provide the needed landscape details and habitat parameters.

## Supporting information

S1 Appendix Mark-release-recapture data of all three butterfly species studied. Given are individuals captured and recaptured per plot and sex of each individual, as well as exact GPS coordinates of each plot.
(XLSX)

S2 Appendix Dispersal data assessed for each of the three butterfly species. (DOCX)
