# Supplementary material for: Mark-release-recapture meets Species Distribution Models: Identifying micro-habitats of grassland butterflies in agricultural landscapes
Source: PLoS One. 2018 Nov 28;13(11):e0207052. doi: 10.1371/journal.pone.0207052 (PMC6261544; doi:10.1371/journal.pone.0207052)
Supplement: S2 Appendix — (DOCX) [file pone.0207052.s002.docx]

Electronic Appendix: Radius of action. Given is the following information for all three species studied: Year of data collection, diameter, perimeter, area and radius covered by the respective individual.

| **Species** | **Year** | **Diameter (m)** | **Perimeter (m)** | **Area (m²)** | **Radius (m)** |
| --- | --- | --- | --- | --- | --- |
| C_arcania | 2015 | 33.9 | 106.5 | 903.1 | 17.0 |
| C_arcania | 2015 | 33.9 | 106.5 | 903.1 | 17.0 |
| C_arcania | 2015 | 26.0 | 81.8 | 531.9 | 13.0 |
| C_arcania | 2015 | 24.3 | 76.4 | 464.7 | 12.2 |
| C_arcania | 2015 | 10.1 | 31.8 | 80.6 | 5.1 |
| C_arcania | 2015 | 39.9 | 125.2 | 1248.3 | 19.9 |
| C_arcania | 2015 | 10.1 | 31.8 | 80.6 | 5.1 |
| C_arcania | 2015 | 34.2 | 107.4 | 917.3 | 17.1 |
| C_arcania | 2015 | 42.7 | 134.3 | 1435.0 | 21.4 |
| C_arcania | 2015 | 26.7 | 83.8 | 558.3 | 13.3 |
| C_arcania | 2015 | 54.7 | 172.0 | 2353.1 | 27.4 |
| C_arcania | 2015 | 13.0 | 40.7 | 131.8 | 6.5 |
| C_arcania | 2015 | 26.7 | 83.8 | 558.3 | 13.3 |
| C_arcania | 2015 | 39.9 | 125.2 | 1247.7 | 19.9 |
| C_arcania | 2015 | 39.9 | 125.2 | 1248.3 | 19.9 |
| C_arcania | 2015 | 30.9 | 97.2 | 751.4 | 15.5 |
| C_arcania | 2015 | 30.9 | 97.2 | 751.4 | 15.5 |
| C_arcania | 2015 | 42.4 | 133.2 | 1411.6 | 21.2 |
| C_arcania | 2015 | 53.3 | 167.3 | 2227.7 | 26.6 |
| C_arcania | 2015 | 62.9 | 197.7 | 3111.5 | 31.5 |
| C_arcania | 2015 | 49.1 | 154.4 | 1896.0 | 24.6 |
| C_arcania | 2015 | 11.3 | 35.6 | 101.0 | 5.7 |
| C_arcania | 2015 | 11.5 | 36.1 | 103.9 | 5.8 |
| C_arcania | 2015 | 10.1 | 31.8 | 80.6 | 5.1 |
| C_arcania | 2015 | 33.9 | 106.5 | 903.1 | 17.0 |
| C_arcania | 2015 | 26.6 | 83.7 | 556.9 | 13.3 |
| C_arcania | 2015 | 23.8 | 74.9 | 446.1 | 11.9 |
| C_arcania | 2015 | 47.1 | 148.1 | 1744.9 | 23.6 |
| C_arcania | 2015 | 38.6 | 121.2 | 1169.3 | 19.3 |
| C_arcania | 2015 | 45.5 | 142.9 | 1624.4 | 22.7 |
| C_arcania | 2015 | 100.9 | 316.9 | 7992.7 | 50.4 |
| C_arcania | 2015 | 16.3 | 51.2 | 208.8 | 8.2 |
| C_arcania | 2015 | 42.4 | 133.2 | 1411.6 | 21.2 |
| C_arcania | 2015 | 75.9 | 238.5 | 4525.8 | 38.0 |
| C_arcania | 2015 | 10.1 | 31.8 | 80.6 | 5.1 |
| C_arcania | 2015 | 104.2 | 327.5 | 8533.2 | 52.1 |
| C_arcania | 2017 | 208.7 | 655.6 | 34207.6 | 104.3 |
| C_arcania | 2017 | 105.6 | 331.7 | 8755.7 | 52.8 |
| C_arcania | 2017 | 161.2 | 506.4 | 20404.9 | 80.6 |
| C_arcania | 2017 | 47.8 | 150.1 | 1792.0 | 23.9 |
| C_arcania | 2017 | 101.8 | 319.8 | 8140.3 | 50.9 |
| C_arcania | 2017 | 173.4 | 544.7 | 23609.4 | 86.7 |
| C_arcania | 2017 | 238.3 | 748.8 | 44616.2 | 119.2 |
| C_arcania | 2017 | 275.8 | 866.4 | 59733.9 | 137.9 |
| C_arcania | 2017 | 87.4 | 274.5 | 5994.8 | 43.7 |
| C_arcania | 2017 | 41.0 | 128.9 | 1323.0 | 20.5 |
| C_arcania | 2017 | 99.8 | 313.4 | 7817.6 | 49.9 |
| C_arcania | 2017 | 75.9 | 238.5 | 4525.7 | 38.0 |
| C_arcania | 2017 | 35.5 | 111.5 | 988.7 | 17.7 |
| C_arcania | 2017 | 41.0 | 128.9 | 1323.0 | 20.5 |
| C_arcania | 2017 | 181.1 | 569.0 | 25768.5 | 90.6 |
| C_arcania | 2017 | 132.3 | 415.6 | 13745.5 | 66.1 |
| C_arcania | 2017 | 55.1 | 173.2 | 2388.4 | 27.6 |
| C_arcania | 2017 | 48.8 | 153.4 | 1872.1 | 24.4 |
| C_arcania | 2017 | 22.6 | 70.9 | 399.5 | 11.3 |
| C_arcania | 2017 | 63.1 | 198.1 | 3124.5 | 31.5 |
| C_arcania | 2017 | 48.8 | 153.4 | 1872.1 | 24.4 |
| C_arcania | 2017 | 69.5 | 218.2 | 3789.3 | 34.7 |
| C_arcania | 2017 | 84.5 | 265.5 | 5608.3 | 42.3 |
| C_arcania | 2017 | 41.8 | 131.4 | 1374.9 | 20.9 |
| C_arcania | 2017 | 38.8 | 121.9 | 1181.7 | 19.4 |
| C_arcania | 2017 | 139.2 | 437.3 | 15216.1 | 69.6 |
| C_arcania | 2017 | 38.8 | 121.9 | 1181.7 | 19.4 |
| C_arcania | 2017 | 156.0 | 490.0 | 19107.9 | 78.0 |
| C_arcania | 2017 | 60.7 | 190.8 | 2898.2 | 30.4 |
| C_arcania | 2017 | 153.5 | 482.4 | 18516.2 | 76.8 |
| C_arcania | 2017 | 56.7 | 178.0 | 2521.8 | 28.3 |
| C_arcania | 2017 | 41.0 | 128.9 | 1323.0 | 20.5 |
| C_arcania | 2017 | 142.6 | 448.1 | 15980.0 | 71.3 |
| C_arcania | 2017 | 47.8 | 150.1 | 1792.0 | 23.9 |
| C_arcania | 2017 | 95.7 | 300.7 | 7195.0 | 47.9 |
| C_arcania | 2017 | 56.7 | 178.0 | 2521.8 | 28.3 |
| C_arcania | 2017 | 50.8 | 159.5 | 2025.6 | 25.4 |
| C_arcania | 2017 | 55.1 | 173.2 | 2388.4 | 27.6 |
| C_arcania | 2017 | 55.1 | 173.2 | 2388.4 | 27.6 |
| C_arcania | 2017 | 55.1 | 173.2 | 2388.4 | 27.6 |
| C_arcania | 2017 | 105.6 | 331.7 | 8755.7 | 52.8 |
| C_arcania | 2017 | 57.6 | 180.9 | 2604.3 | 28.8 |
| C_arcania | 2017 | 155.7 | 489.0 | 19031.4 | 77.8 |
| C_arcania | 2017 | 100.3 | 315.2 | 7904.0 | 50.2 |
| C_arcania | 2017 | 117.7 | 369.8 | 10885.2 | 58.9 |
| C_arcania | 2017 | 155.7 | 489.0 | 19031.4 | 77.8 |
| C_arcania | 2017 | 22.6 | 70.9 | 399.5 | 11.3 |
| E_medusa | 2015 | 104.2 | 327.5 | 8533.2 | 52.1 |
| E_medusa | 2015 | 49.1 | 154.4 | 1896.0 | 24.6 |
| E_medusa | 2015 | 23.9 | 75.0 | 447.9 | 11.9 |
| E_medusa | 2015 | 27.4 | 86.2 | 591.7 | 13.7 |
| E_medusa | 2015 | 37.9 | 119.0 | 1127.5 | 18.9 |
| E_medusa | 2015 | 27.4 | 86.2 | 591.7 | 13.7 |
| E_medusa | 2015 | 47.7 | 150.0 | 1790.3 | 23.9 |
| E_medusa | 2015 | 30.7 | 96.5 | 741.5 | 15.4 |
| E_medusa | 2015 | 34.1 | 107.2 | 913.8 | 17.1 |
| E_medusa | 2015 | 30.7 | 96.5 | 741.5 | 15.4 |
| E_medusa | 2015 | 53.3 | 167.3 | 2227.7 | 26.6 |
| E_medusa | 2015 | 16.3 | 51.1 | 208.2 | 8.1 |
| E_medusa | 2015 | 45.0 | 141.4 | 1590.9 | 22.5 |
| E_medusa | 2017 | 52.0 | 163.3 | 2123.1 | 26.0 |
| E_medusa | 2017 | 281.5 | 884.4 | 62244.2 | 140.8 |
| E_medusa | 2017 | 140.2 | 440.5 | 15441.3 | 70.1 |
| E_medusa | 2017 | 92.7 | 291.1 | 6742.5 | 46.3 |
| E_medusa | 2017 | 114.1 | 358.6 | 10231.0 | 57.1 |
| E_medusa | 2017 | 114.1 | 358.6 | 10231.0 | 57.1 |
| E_medusa | 2017 | 114.1 | 358.6 | 10231.0 | 57.1 |
| E_medusa | 2017 | 99.8 | 313.4 | 7817.6 | 49.9 |
| E_medusa | 2017 | 99.8 | 313.4 | 7817.6 | 49.9 |
| E_medusa | 2017 | 95.1 | 298.8 | 7105.9 | 47.6 |
| E_medusa | 2017 | 148.8 | 467.4 | 17384.0 | 74.4 |
| E_medusa | 2017 | 184.4 | 579.4 | 26715.4 | 92.2 |
| E_medusa | 2017 | 105.6 | 331.7 | 8755.7 | 52.8 |
| E_medusa | 2017 | 200.4 | 629.6 | 31540.7 | 100.2 |
| E_medusa | 2017 | 126.7 | 397.9 | 12600.1 | 63.3 |
| E_medusa | 2017 | 164.7 | 517.5 | 21314.2 | 82.4 |
| E_medusa | 2017 | 254.3 | 798.8 | 50772.8 | 127.1 |
| E_medusa | 2017 | 151.0 | 474.2 | 17897.0 | 75.5 |
| E_medusa | 2017 | 73.5 | 231.1 | 4248.6 | 36.8 |
| E_medusa | 2017 | 193.9 | 609.0 | 29515.4 | 96.9 |
| E_medusa | 2017 | 97.0 | 304.6 | 7385.2 | 48.5 |
| E_medusa | 2017 | 83.3 | 261.8 | 5452.1 | 41.7 |
| E_medusa | 2017 | 41.8 | 131.4 | 1374.9 | 20.9 |
| E_medusa | 2017 | 270.4 | 849.5 | 57433.0 | 135.2 |
| E_medusa | 2017 | 41.8 | 131.4 | 1374.9 | 20.9 |
| E_medusa | 2017 | 299.1 | 939.6 | 70253.1 | 149.5 |
| E_medusa | 2017 | 65.4 | 205.6 | 3362.5 | 32.7 |
| E_medusa | 2017 | 35.9 | 112.9 | 1013.9 | 18.0 |
| E_medusa | 2017 | 56.7 | 178.0 | 2521.8 | 28.3 |
| E_medusa | 2017 | 95.7 | 300.7 | 7195.0 | 47.9 |
| E_medusa | 2017 | 56.7 | 178.0 | 2521.8 | 28.3 |
| E_medusa | 2017 | 167.8 | 527.1 | 22110.5 | 83.9 |
| E_medusa | 2017 | 96.1 | 301.9 | 7254.1 | 48.1 |
| E_medusa | 2017 | 48.8 | 153.4 | 1872.1 | 24.4 |
| E_medusa | 2017 | 63.1 | 198.1 | 3124.5 | 31.5 |
| E_medusa | 2017 | 181.1 | 569.0 | 25768.5 | 90.6 |
| E_medusa | 2017 | 69.2 | 217.5 | 3763.2 | 34.6 |
| E_medusa | 2017 | 52.6 | 165.4 | 2177.1 | 26.3 |
| E_medusa | 2017 | 55.1 | 173.2 | 2388.4 | 27.6 |
| E_medusa | 2017 | 67.4 | 211.7 | 3567.6 | 33.7 |
| E_medusa | 2017 | 55.1 | 173.2 | 2388.4 | 27.6 |
| E_medusa | 2017 | 189.8 | 596.4 | 28306.4 | 94.9 |
| E_medusa | 2017 | 149.2 | 468.6 | 17476.2 | 74.6 |
| E_medusa | 2017 | 60.7 | 190.8 | 2898.2 | 30.4 |
| E_medusa | 2017 | 85.0 | 267.1 | 5677.0 | 42.5 |
| E_medusa | 2017 | 101.0 | 317.2 | 8004.4 | 50.5 |
| E_medusa | 2017 | 97.0 | 304.6 | 7385.2 | 48.5 |
| E_medusa | 2017 | 56.1 | 176.2 | 2470.7 | 28.0 |
| E_medusa | 2017 | 41.8 | 131.4 | 1374.9 | 20.9 |
| E_medusa | 2017 | 97.0 | 304.6 | 7385.2 | 48.5 |
| E_medusa | 2017 | 97.0 | 304.6 | 7385.2 | 48.5 |
| E_medusa | 2017 | 97.0 | 304.6 | 7385.2 | 48.5 |
| E_medusa | 2017 | 97.0 | 304.6 | 7385.2 | 48.5 |
| E_medusa | 2017 | 97.0 | 304.6 | 7385.2 | 48.5 |
| E_medusa | 2017 | 97.0 | 304.6 | 7385.2 | 48.5 |
| E_medusa | 2017 | 97.0 | 304.6 | 7385.2 | 48.5 |
| E_medusa | 2017 | 97.0 | 304.6 | 7385.2 | 48.5 |
| E_medusa | 2017 | 97.0 | 304.6 | 7385.2 | 48.5 |
| E_medusa | 2017 | 135.1 | 424.5 | 14338.9 | 67.6 |
| E_medusa | 2017 | 164.7 | 517.5 | 21314.2 | 82.4 |
| E_medusa | 2017 | 266.4 | 836.8 | 55718.9 | 133.2 |
| E_medusa | 2017 | 241.8 | 759.5 | 45905.0 | 120.9 |
| E_medusa | 2017 | 247.2 | 776.7 | 48007.5 | 123.6 |
| E_medusa | 2017 | 37.6 | 118.1 | 1109.3 | 18.8 |
| E_medusa | 2017 | 92.7 | 291.1 | 6742.5 | 46.3 |
| E_medusa | 2017 | 99.8 | 313.4 | 7817.6 | 49.9 |
| E_medusa | 2017 | 95.1 | 298.8 | 7105.9 | 47.6 |
| E_medusa | 2017 | 90.3 | 283.7 | 6403.2 | 45.1 |
| E_medusa | 2017 | 73.3 | 230.2 | 4218.3 | 36.6 |
| E_medusa | 2017 | 47.8 | 150.1 | 1792.0 | 23.9 |
| E_medusa | 2017 | 260.1 | 817.0 | 53115.3 | 130.0 |
| E_medusa | 2017 | 135.1 | 424.5 | 14338.9 | 67.6 |
| E_medusa | 2017 | 135.1 | 424.5 | 14338.9 | 67.6 |
| E_medusa | 2017 | 274.4 | 861.9 | 59120.9 | 137.2 |
| E_medusa | 2017 | 31.1 | 97.9 | 762.0 | 15.6 |
| E_medusa | 2017 | 57.6 | 180.9 | 2604.3 | 28.8 |
| E_medusa | 2017 | 50.8 | 159.5 | 2025.6 | 25.4 |
| E_medusa | 2017 | 294.5 | 925.1 | 68106.6 | 147.2 |
| E_medusa | 2017 | 55.1 | 173.2 | 2388.4 | 27.6 |
| E_medusa | 2017 | 42.1 | 132.4 | 1394.9 | 21.1 |
| E_medusa | 2017 | 42.1 | 132.4 | 1394.9 | 21.1 |
| E_medusa | 2017 | 42.1 | 132.4 | 1394.9 | 21.1 |
| E_medusa | 2017 | 121.0 | 380.1 | 11494.4 | 60.5 |
| E_medusa | 2017 | 121.0 | 380.1 | 11494.4 | 60.5 |
| E_medusa | 2017 | 121.0 | 380.1 | 11494.4 | 60.5 |
| E_medusa | 2017 | 111.6 | 350.6 | 9779.9 | 55.8 |
| M_galathea | 2017 | 278.7 | 875.6 | 61008.1 | 139.4 |
| M_galathea | 2017 | 95.1 | 298.8 | 7105.9 | 47.6 |
| M_galathea | 2017 | 212.9 | 668.8 | 35589.3 | 106.4 |
| M_galathea | 2017 | 157.7 | 495.5 | 19535.4 | 78.9 |
| M_galathea | 2017 | 53.9 | 169.5 | 2285.0 | 27.0 |
| M_galathea | 2017 | 101.9 | 320.0 | 8148.5 | 50.9 |
| M_galathea | 2017 | 73.3 | 230.2 | 4218.3 | 36.6 |
| M_galathea | 2017 | 121.3 | 381.1 | 11560.6 | 60.7 |
| M_galathea | 2017 | 167.3 | 525.6 | 21987.5 | 83.7 |
| M_galathea | 2017 | 117.7 | 369.8 | 10885.2 | 58.9 |
| M_galathea | 2017 | 82.8 | 260.1 | 5385.1 | 41.4 |
| M_galathea | 2017 | 257.9 | 810.3 | 52244.7 | 129.0 |
| M_galathea | 2017 | 257.0 | 807.3 | 51859.9 | 128.5 |
| M_galathea | 2017 | 173.4 | 544.7 | 23609.4 | 86.7 |
| M_galathea | 2017 | 123.5 | 388.1 | 11983.1 | 61.8 |
| M_galathea | 2017 | 251.7 | 790.6 | 49742.5 | 125.8 |
| M_galathea | 2017 | 208.7 | 655.6 | 34207.6 | 104.3 |
| M_galathea | 2017 | 38.8 | 121.9 | 1181.7 | 19.4 |
| M_galathea | 2017 | 66.3 | 208.2 | 3450.1 | 33.1 |
| M_galathea | 2017 | 135.7 | 426.3 | 14458.5 | 67.8 |
| M_galathea | 2017 | 142.8 | 448.7 | 16023.9 | 71.4 |
| M_galathea | 2017 | 114.1 | 358.6 | 10231.0 | 57.1 |
| M_galathea | 2017 | 186.9 | 587.2 | 27438.5 | 93.5 |
| M_galathea | 2017 | 120.6 | 379.0 | 11430.1 | 60.3 |
| M_galathea | 2017 | 52.0 | 163.3 | 2123.1 | 26.0 |
| M_galathea | 2017 | 294.4 | 925.0 | 68084.2 | 147.2 |
| M_galathea | 2017 | 114.1 | 358.6 | 10231.0 | 57.1 |
| M_galathea | 2017 | 140.5 | 441.3 | 15496.2 | 70.2 |
| M_galathea | 2017 | 193.9 | 609.0 | 29515.4 | 96.9 |
| M_galathea | 2017 | 165.8 | 520.9 | 21588.4 | 82.9 |
| M_galathea | 2017 | 140.2 | 440.5 | 15441.3 | 70.1 |
| M_galathea | 2017 | 139.2 | 437.3 | 15216.1 | 69.6 |
| M_galathea | 2017 | 267.8 | 841.3 | 56322.1 | 133.9 |
| M_galathea | 2017 | 100.4 | 315.4 | 7917.3 | 50.2 |
| M_galathea | 2017 | 38.8 | 121.9 | 1181.7 | 19.4 |
| M_galathea | 2017 | 257.5 | 808.9 | 52066.0 | 128.7 |
| M_galathea | 2017 | 68.1 | 213.8 | 3638.1 | 34.0 |
| M_galathea | 2017 | 35.5 | 111.5 | 988.7 | 17.7 |
| M_galathea | 2017 | 117.7 | 369.8 | 10885.2 | 58.9 |
| M_galathea | 2017 | 140.5 | 441.3 | 15496.2 | 70.2 |
| M_galathea | 2017 | 204.1 | 641.3 | 32731.0 | 102.1 |
| M_galathea | 2017 | 121.0 | 380.1 | 11494.4 | 60.5 |
| M_galathea | 2017 | 183.5 | 576.6 | 26453.6 | 91.8 |
| M_galathea | 2017 | 157.8 | 495.7 | 19550.2 | 78.9 |
| M_galathea | 2017 | 244.9 | 769.2 | 47088.5 | 122.4 |
| M_galathea | 2017 | 69.5 | 218.2 | 3789.3 | 34.7 |
| M_galathea | 2017 | 99.7 | 313.3 | 7813.1 | 49.9 |
| M_galathea | 2017 | 73.3 | 230.2 | 4218.3 | 36.6 |
| M_galathea | 2017 | 121.0 | 380.1 | 11494.4 | 60.5 |
| M_galathea | 2017 | 108.1 | 339.7 | 9182.1 | 54.1 |
| M_galathea | 2017 | 142.6 | 448.1 | 15980.0 | 71.3 |
| M_galathea | 2017 | 142.6 | 448.1 | 15980.0 | 71.3 |
| M_galathea | 2017 | 167.8 | 527.1 | 22111.6 | 83.9 |
| M_galathea | 2017 | 149.2 | 468.6 | 17476.2 | 74.6 |
| M_galathea | 2017 | 155.7 | 489.0 | 19031.4 | 77.8 |
| M_galathea | 2017 | 73.5 | 231.1 | 4248.6 | 36.8 |
| M_galathea | 2017 | 73.5 | 231.1 | 4248.6 | 36.8 |
| M_galathea | 2017 | 41.0 | 128.9 | 1323.0 | 20.5 |
| M_galathea | 2017 | 127.1 | 399.3 | 12690.4 | 63.6 |
| M_galathea | 2017 | 149.2 | 468.6 | 17476.2 | 74.6 |
| M_galathea | 2017 | 194.7 | 611.5 | 29760.6 | 97.3 |
| M_galathea | 2017 | 281.5 | 884.4 | 62244.2 | 140.8 |
| M_galathea | 2017 | 65.4 | 205.6 | 3362.5 | 32.7 |
| M_galathea | 2017 | 123.5 | 388.1 | 11983.1 | 61.8 |
| M_galathea | 2017 | 123.5 | 388.1 | 11983.1 | 61.8 |
| M_galathea | 2017 | 125.9 | 395.4 | 12440.0 | 62.9 |
| M_galathea | 2017 | 90.7 | 284.8 | 6456.1 | 45.3 |
| M_galathea | 2017 | 90.7 | 284.8 | 6456.1 | 45.3 |
| M_galathea | 2017 | 90.7 | 284.8 | 6456.1 | 45.3 |
| M_galathea | 2017 | 257.9 | 810.3 | 52244.7 | 129.0 |
| M_galathea | 2017 | 90.7 | 284.8 | 6456.1 | 45.3 |
| M_galathea | 2017 | 56.1 | 176.2 | 2470.7 | 28.0 |
| M_galathea | 2017 | 73.5 | 231.1 | 4248.6 | 36.8 |
| M_galathea | 2017 | 54.1 | 170.0 | 2300.3 | 27.1 |
| M_galathea | 2017 | 114.0 | 358.3 | 10214.2 | 57.0 |
| M_galathea | 2017 | 186.9 | 587.2 | 27438.5 | 93.5 |
| M_galathea | 2017 | 278.7 | 875.6 | 61008.1 | 139.4 |
| M_galathea | 2017 | 59.8 | 187.7 | 2804.4 | 29.9 |
| M_galathea | 2017 | 167.8 | 527.1 | 22111.6 | 83.9 |
| M_galathea | 2017 | 123.5 | 388.1 | 11983.1 | 61.8 |
| M_galathea | 2017 | 66.3 | 208.2 | 3450.1 | 33.1 |
| M_galathea | 2017 | 120.6 | 379.0 | 11430.1 | 60.3 |
| M_galathea | 2017 | 139.2 | 437.3 | 15216.1 | 69.6 |
| M_galathea | 2017 | 38.8 | 121.9 | 1181.7 | 19.4 |
| M_galathea | 2017 | 173.4 | 544.7 | 23609.4 | 86.7 |
| M_galathea | 2017 | 173.4 | 544.7 | 23609.4 | 86.7 |
| M_galathea | 2017 | 203.2 | 638.2 | 32415.3 | 101.6 |
| M_galathea | 2017 | 186.9 | 587.2 | 27438.5 | 93.5 |
| M_galathea | 2017 | 201.5 | 632.9 | 31877.9 | 100.7 |
| M_galathea | 2017 | 135.8 | 426.5 | 14473.8 | 67.9 |
| M_galathea | 2017 | 68.1 | 213.8 | 3638.1 | 34.0 |
| M_galathea | 2017 | 241.8 | 759.5 | 45905.0 | 120.9 |
| M_galathea | 2017 | 67.3 | 211.6 | 3562.1 | 33.7 |
| M_galathea | 2017 | 120.6 | 379.0 | 11430.1 | 60.3 |
| M_galathea | 2017 | 281.5 | 884.4 | 62244.2 | 140.8 |
| M_galathea | 2017 | 146.3 | 459.7 | 16820.1 | 73.2 |
| M_galathea | 2017 | 294.4 | 925.0 | 68084.2 | 147.2 |
| M_galathea | 2017 | 146.3 | 459.7 | 16820.1 | 73.2 |
| M_galathea | 2017 | 52.0 | 163.3 | 2123.1 | 26.0 |
